# Evaluation of Sleep Quality and Related Factors in Community-Dwelling Adults: Ardakan Cohort Study on Aging (ACSA)

**DOI:** 10.34172/jrhs.2023.126

**Published:** 2023-09-29

**Authors:** Ahmad Delbari, Fatemeh Ghavidel, Mohammad Bidkhori, Mohammad Saatchi, Yadollah Abolfathi Momtaz, Sara Efati, Elham Hooshmand

**Affiliations:** ^1^Iranian Research Center on Aging, University of Social Welfare and Rehabilitation Sciences, Tehran, Iran; ^2^Department of Biostatistics and Epidemiology, School of Rehabilitation, University of Social Welfare and Rehabilitation Science, Tehran, Iran; ^3^Health in Emergency and Disaster Research Center, University of Social Welfare and Rehabilitation Sciences, Tehran, Iran

**Keywords:** Sleep quality, Sleep duration, Geriatric assessment, Depression, Iran

## Abstract

**Background:** Sleep is a necessary physiological process that affects health. The current study aimed to evaluate sleep quality (SQ) and the related factors in Iranian community-dwelling adults.

**Study Design:** A cross-sectional study.

**Methods:** Population-based cross-sectional data from the first wave of the Ardakan cohort study on aging (ACSA) were analyzed. The analytical sample consisted of 5197 community-dwelling adults aged≥50. All data were collected by trained staff. Pittsburgh Sleep Quality Index (PSQI) scores were used to measure SQ. Univariable and multivariable logistic regression were used to identify predictors of SQ.

**Results:** The mean age of the participants was 62.22±7.7 years, and 51.8% were female. About threequarters of them (76.36%) were found to have poor SQ (PSQI score≥5). Multivariable logistic regression analysis revealed a relationship between SQ and gender, education, work, and financial level. Furthermore, SQ was found to be associated with self-reported health and physical activity. Regarding comorbidity, SQ had a significant relationship with cardiovascular diseases, musculoskeletal diseases, depression, and anxiety (*P*<0.05).

**Conclusion:** The prevalence of poor SQ was high in these community-dwelling adults in Iran. These findings highlight the importance of intervention programs for sleep hygiene education and screening for middle-aged and older adults.

## Background

 Aging is a natural biological process, and the global population is aging rapidly. According to the World Health Organization (WHO), one in six people will be aged over 60 by 2030, and two-thirds of the world’s older adult population will be living in low- and middle-income countries by 2050.^1^ Furthermore, studies predict that the older adult population in Iran, similar to other developing countries, will experience the fastest growth compared to other age groups, and the phenomenon of population aging in Iran is inevitable.^2^

 Increasing age can lead to changes in sleep quality (SQ) and patterns, an increase in the total amount of time spent in bed, and a greater percentage of time in stage I sleep. Alternatively, aging is associated with a decrease in sleep duration, a longer latency to Rapid eye movement (REM) sleep, and a decrease in slow-wave sleep percentage.^3,4^ Consequently, older adults may experience frequent complaints due to changes in their sleep patterns and quality.

 Poor SQ increases the risk of various physical and mental health problems.^5,6^ Numerous studies demonstrated that poor SQ is associated with an increased risk of falls, cognitive disorders, dementia, reduced concentration, and mortality-related outcomes.^7-12^ It is important to note that alterations in sleep patterns during the aging process can adversely affect the quality of life.^13-15^

 Reduced SQ in adults has been associated with several factors. Research has linked poor SQ to Alzheimer’s disease, chronic diseases, musculoskeletal pain, osteoporosis, diabetes, and heart disease in older adults.^16-19^ Lifestyle factors, including physical activity, can also affect SQ.^20^ Additionally, depression and anxiety are important psychological factors that have been linked to poor SQ.^21,22^

 Despite the numerous studies on SQ in developed countries, developing countries require more investigation on SQ and its determinants. In addition, to the best of our knowledge, no study has evaluated SQ in Iranian community-dwelling adults. Given the potential psychological burden and adverse effects of poor SQ in older adults, large-scale data are needed to evaluate this issue. The present study aimed to address this gap by examining the prevalence of poor SQ and related factors using representative data from a large sample of adults aged 50 years and older.

## Methods

###  Study Participants

 The data for this study were collected during the first wave of the Ardakan cohort study on aging (ACSA) between 2020 and 2022. ACSA is a subset of the Iranian longitudinal study on aging (IRLSA), the details of which have been published elsewhere.^23^ The ACSA study is a longitudinal cohort that began in 2020 to examine different aspects of the health of middle-aged and older adult residents of Ardakan in the central part of Iran, located in the Yazd province. The ACSA used a multi-stage stratified random sampling method to select a sample of over 5000 individuals aged 50 years or older. This cohort included citizens who were 50 years or older and residing in Ardakan. Participants with a diagnosis of dementia, deafness, blindness, mental or psychological disorders such as mental retardation or psychosis, paralysis, and inability to understand and answer the study questions were excluded. All individuals with available sleep data were included in the present study.

###  Sleep Quality 

 The SQ was assessed using the Pittsburgh Sleep Quality Index (PSQI) questionnaire, which has been validated in the Iranian population.^24^ The PSQI is a standardized self-administered questionnaire that assesses SQ for the past month. The questionnaire consists of 19 items that are classified into seven categories: subjective sleep quality, sleep latency, sleep duration, habitual sleep efficiency, sleep disturbances, use of sleep medication, and daytime dysfunction. Each component is scored on a scale of 0 to 3, with higher scores indicating worse SQ. The sum of the scores for the seven components yields the global PSQI score, which ranges from 0 to 21. A global PSQI score higher than 5 indicates poor SQ, while lower scores suggest good SQ.

###  Covariates

 To measure depression, the Center for Epidemiological Studies Depression Scale (CES-D) questionnaire was used, which was validated and reliable in the Iranian population by Malakouti et al.^25^ The CES-D is a self-report questionnaire consisting of 10 items, designed to assess depressive feelings and behaviors experienced within the past week. Each symptom is scored on a 1 to 4 Likert scale, where 1 means “not at all,” 2 means “sometimes,” 3 means “usually,” and 4 means “yes, almost always”. The total score ranges from 0 to 30, based on the sum of the scores of all the items. Higher scores reflect more depressive symptoms.

 The Hospital Anxiety and Depression Scale (HADS) is an internationally standardized and reliable instrument used to measure depression and anxiety. It comprises two subscales: depression (HADS-D) and anxiety (HADS-A).^26^ For this study, only the HADS-A subscale was used to assess anxiety. HADS-A consists of seven items (with four-choice answers) and a score ranging from 0 to 3. Scores between 0-7 are considered normal anxiety, 7-14 are classified as borderline anxiety, and 14-21 indicate abnormal levels of anxiety. The validity of the questionnaire was confirmed in the Iranian population by Montazeri et al.^27^

 The level of physical activity was assessed using the Physical Activity Scale for the Elderly (PASE).^28^ The PASE is a self-report questionnaire that measures the level of physical activity over the past week. The overall PASE score ranges from 0 to 400 or more. It comprises 10 items that are classified into three categories: leisure time activity, household activity, and work-related activity.^29^

 To examine socioeconomic status, this study considered indicators such as age, gender, marital status, education, employment status, and economic status. Both self-rated health and economic status were evaluated by asking the question, “How would you describe your overall health/economic status?” on a five-point scale. The participants were classified into four categories according to body mass index (BMI): underweight (BMI ≤ 18 kg/m^2^), normal (18.5 kg/m^2^ ≤ BMI < 25 kg/m^2^), overweight (BMI ≥ 25 and < 30 kg/m^2^), and obese (BMI ≥ 30 kg/m^2^). The living arrangements were evaluated based on their response to the question “With whom do you live most of the time?”.

 Comorbidities were assessed via self-report and categorized into four groups: respiratory diseases (e.g., chronic obstructive pulmonary disease, asthma, and chronic bronchitis), cardiovascular diseases (e.g., heart attack, heart failure, angina pectoris, and hypertension), musculoskeletal diseases (e.g., arthritis and osteoarthritis), and metabolic diseases (e.g., type 2 diabetes, hyperthyroidism, and hypothyroidism).

###  Ethical Considerations

 This study was conducted in accordance with the ethical principles of the University of Welfare and Rehabilitation Sciences and received approval from the Ethics Committee (code of ethics: IR.USWR.REC.1394.490). All participants provided informed consent after being informed about the study’s objectives and methods.

###  Data Analysis

 First, descriptive analysis was performed for continuous variables using mean and standard deviation and for categorical variables using frequency and percentage. The outcome variable was entered into the model as a binary variable. A logistic regression model was employed in two steps to evaluate the adjusted association of variables with SQ. Firstly, the univariable model was used to assess the unadjusted association of each independent variable with SQ, with a significance threshold of 0.2 (α: 0.2). Then, a multivariable model was fitted using a stepwise backward approach to evaluate the independent effects of each variable adjusted for other covariates, with a significance threshold of 0.05 (α: 0.05). In the present study, reference levels of interest were selected for multilevel variables such as education level and employment were chosen based on the sample size of each level. In addition, for variables such as economic status and self-reported health, the middle level was used as a reference to provide a more balanced view of the variable’s distribution and facilitate meaningful interpretation of the results. STATA Statistical Software version 15 (Stata-Corp., 2017) was used to perform all data analyses.

## Results

 Of the 5224 people who completed the PSQI questionnaire by the time of this study, 5197 (99%) had demographic information available and were included in the present study. The mean age of participants was 62.22 (7.7) years, of whom 51.86% were female. The majority of the participants were married (90.94%) and lived with their spouses (94.51%). Out of all the participants, 3969 (76.36%) had low SQ, and most of them (57%) were women. The mean score of PASE was 129.86 (80.89) for the poor SQ group and 156.69 (94.88) for the good SQ group. Half of the individuals with poor SQ reported cardiovascular diseases. Additionally, approximately 32% of those with poor SQ reported abnormal or borderline anxiety, while about 22% experienced depressive feelings in the past week. Further details are presented in Table 1.

**Table 1:** Baseline characteristics of population under study based on SQ

**Variables**	**Total**		**Poor SQ**		**Good SQ**	
**Categorical variables**	**Number**	**Percent**	**Number**	**Percent**	**Number**	**Percent**
Gender						
Female	2695	51.86	2295	57.82	400	32.57
Male	2502	48.14	1674	42.18	828	67.43
Marital status						
Unmarried	471	9.00	416	10.48	55	4.48
Married	4726	90.94	3553	89.52	1173	95.52
Education						
No formal education	2478	14.16	620	15.67	114	9.29
Elementary	2478	47.81	1980	50.05	498	40.59
Middle	756	14.59	559	14.13	197	16.06
High School	623	12.02	435	11.00	188	15.32
College	594	11.42	362	9.15	230	18.74
Job status						
Unemployed/retired	3360	64.83	2604	65.82	756	61.62
Working	1106	21.34	756	19.11	350	28.52
Other/housekeeper	717	13.83	596	15.07	121	9.86
Self-expressed financial level						
Highest	22	0.44	15	0.40	7	0.57
High	351	7.00	236	6.23	115	9.43
Middle	2463	49.17	1807	47.69	656	53.77
Low	1244	24.84	968	25.55	276	22.62
Lowest	929	18.55	763	20.13	166	13.61
Self-rated health						
Extremely good	205	3.96	104	2.63	101	8.23
Good	1519	29.35	975	24.70	544	44.34
Fair	2971	57.42	2443	61.62	538	43.85
Poor	396	7.65	354	8.97	42	3.42
Extremely Poor	84	1.62	82	2.08	2	0.16
Living arrangements						
Alone	279	5.49	247	6.37	32	2.66
With others	4800	94.51	3631	93.63	1169	97.34
Respiratory diseases	339	6.52	0.925	0.42	55	4.47
Cardiovascular diseases	2625	50.49	2086	52.56	539	43.82
Musculoskeletal diseases	1652	31.78	1412	35.58	240	19.51
Metabolic diseases	1997	38.41	1618	40.77	379	30.81
Anxiety						
Normal	3686	74.26	2530	67.70	1156	94.22
Borderline	693	13.96	645	17.26	48	3.91
Abnormal	585	11.78	562	15.04	23	1.87
Depression						
Normal	4086	82.73	2887	77.71	1199	97.96
Abnormal	853	17.27	828	22.29	25	2.04
**Continuous variables**	**Mean**	**SD**	**Mean**	**SD**	**Mean**	**SD**
Age	62.22	7.70	62.35	7.80	61.84	7.60
Number of rooms/people in the house	0.922	0.42	0.925	0.42	0.922	0.42
Physical activity	136.16	85.17	129.86	80.89	156.69	94.88

*Note.* SQ: Sleep quality; SD: Standard deviation.

Table 2 presents the descriptive indices of the SQ components. The mean global PSQI score was 7.90 (4.25). Among the different components, the “sleep efficiency” component had the highest mean score (2.11), while the “use of sleep medication” component had the lowest mean score among the subjects (0.67).

**Table 2:** Components of PSQI in participants

	**Range**	**Mean**	**SD**
Component 1: Subjective sleep quality	0-3	0.91	0.64
Component 2: Sleep latency	0-3	1.58	1.11
Component 3: Sleep duration	0-3	1.74	1.16
Component 4: Sleep efficiency	0-3	2.11	1.65
Component 5: Sleep disturbance	0-3	0.81	0.53
Component 6: Use of sleep medication	0-3	0.67	1.22
Component 7: Daytime dysfunction	0-3	1.13	1.40
Global PSQI score	0-21	7.90	4.26

*Note.* PSQI: Pittsburgh sleep quality index; SD: Standard deviation.

Table 3 displays the results of the univariable logistic regression analysis. The findings revealed that the odds of poor SQ were 65% higher among females than males (odds ratio [OR] = 0.35, 95% confidence interval [CI]: 0.30 to 0.40). Similarly, single people had nearly 60% higher odds of poor SQ than married individuals (OR = 0.41, 95% CI: 0.30 to 0.54). Participants who lived alone were 60% more likely to have poor SQ than those who lived with others (OR = 0.40, 95% CI: 0.28 to 0.58). Additionally, those who rated their health as “good” or “extremely good” had better SQ than those who reported “fair” health status (*P* < 0.001). The results also revealed that cardiovascular disease (OR = 1.42, 95% CI: 1.24 to 1.61), musculoskeletal disease (OR = 2.27, 95% CI: 1.95 to 2.66), metabolic syndrome (OR = 1.54, 95% CI: 1.35 to 1.77), respiratory diseases (OR = 1.64, 95% CI: 1.22 to 2.21), and depression (OR = 13.75, 95% CI: 9.18 to 20.8) were all associated with poor SQ.

**Table 3:** Logistic Regression of Sleep Quality and Related Factors

**Variables**	**Unadjusted OR (95% CI)**	***P***** value**	**Adjusted OR (95% CI)**	***P***** value**
Age (per one year)	1.01 (1.00, 1.07)	0.036	-	-
Gender				
Female	1.00		1.00	
Male	0.35 (0.30, 0.40)	0.001	0.55 (0.46, 0.66)	0.001
Marital status				
Unmarried	1.00			
Married	0.41 (0.30, 0.54)	0.001	-	-
Education level				
Elementary	1.00		1.00	
no-formal education	1.36 (1.09, 1.70)	0.011	1.14 (0.89, 1.46)	0.267
Middle	0.71 (0.59, 0.86)	0.001	0.97 (0.78, 1.20)	0.789
High School	0.58 (0.47, 0.70)	0.001	0.92 (0.74, 1.16)	0.528
College	0.39 (0.32, 0.47)	0.001	0.73 (0.59, 0.92)	0.008
Employment status				
Unemployed/retired	1.00		1.00	
Working	0.63 (0.54, 0.72)	0.001	0.67 (0.57, 0.80)	0.001
Other/housekeeper	1.44 (1.16, 1.70)	0.001	0.83 (0.64, 1.07)	0.157
Self-rated health				
Fair	1.00		1.00	
Extremely Good	0.22 (0.17, 0.30)	0.001	0.39 (0.28, 0.53)	0.001
Good	0.39 (0.34, 0.45)	0.001	0.60 (0.51, 0.70)	0.001
Poor	1.87 (1.34, 2.61)	0.001	1.14 (0.79, 1.66)	0.464
Extremely Poor	9.04 (2.21, 36.8)	0.001	2.90 (0.67, 12.42)	0.151
Self-expressed financial level				
Middle	1.00		1.00	
Highest	0.77 (0.31, 1.90)	0.558	1.62 (0.62, 4.21)	0.319
High	0.74 (0.58, 0.93)	0.016	1.08 (0.82, 1.41)	0.572
Low	1.27 (1.08, 1.49)	0.003	1.24 (1.03, 1.48)	0.017
Lowest	1.66 (1.36, 1.99)	0.001	1.27 (1.03, 1.58)	0.025
Living arrangements				
Alone	1.00			
With others	0.40 (0.28, 0.58)	0.001	-	-
Number of rooms/people in the house	1.07 (0.92, 1.26)	0.354	-	-
Respiratory diseases				
No	1.00		-	
Yes	1.64 (1.22, 2.21)	0.001	-	-
Cardiovascular diseases				0.032
No	1.00		1.00	
Yes	1.42 (1.24, 1.61)	0.001	1.17 (1.01, 1.35)	
Musculoskeletal diseases				
No	1.00		1.00	
Yes	2.27 (1.95, 2.66)	0.001	1.47 (1.23, 1.75)	0.001
Metabolic diseases				
No	1.00		-	
Yes	1.54 (1.35, 1.77)	0.001	-	-
Anxiety				
Normal	1.00		1.00	
Borderline	6.13 (4.45, 8.29)	0.001	3.36 (2.42, 4.59)	0.001
Abnormal	11.16 (7.31, 17.0)	0.001	3.44 (2.14, 5.48)	0.001
Depression				
Normal	1.00		1.00	
Abnormal	13.75 (9.18, 20.8)	0.001	4.61 (2.95, 7.24)	0.001
PASE	0.99 (0.99, 0.99)	0.001	0.99 (0.998, 0.999)	0.009

*Note.* PASE: Physical activity of elderly; OR: Odds ratio; CI: Confidence interval.

Table 3 presents the results of the multivariable logistic regression models adjusted for covariates. The findings of the study indicated a significant association between poor SQ and gender among participants, with females having 45% higher odds of poor SQ than males, even after adjusting for potential covariates (OR = 0.55, 95% CI: 0.46 to 0.66). The odds of having poor SQ were 33% higher for subjects who were working than for those who were not working or retired (OR = 0.67, 95% CI: 0.57 to 0.80). Furthermore, compared to participants who reported “fair” self-health-related status, those who reported “good” had 40% lower odds of poor SQ (OR = 0.60, 95 % CI: 0.51 to 0.70). Similarly, those who reported “extremely good” self-health-related status had 61% lower odds of poor SQ (OR = 0.39, 95% CI: 0.67 to12.42).

 The findings showed a significant relationship between physical activity and SQ, where a one-unit increase in PASE score reduces the odds of poor SQ by one percent (OR = 0.99, 95% CI:0.998 to 0.999). Additionally, the odds of poor SQ were 4.61 times higher in individuals with depression than in non-depressed individuals (95% CI: 2.95 to 7.24). Similarly, individuals with borderline and abnormal anxiety had higher odds of poor SQ than normal individuals (OR = 3.36, 95% CI: 2.42 to 4.59 and OR = 3.44, 95% CI: 2.14 to 5.48, respectively). Compared to participants who reported cardiovascular and musculoskeletal diseases, those who did not report these conditions had better SQ (OR = 1.17, 95% CI: 1.01 to 1.35 and OR = 1.47, 95% CI: 1.23 to 1.75, respectively).

## Discussion

 This study aimed to assess SQ and related factors in Iranian individuals aged 50 years and above. To the best of the authors’ knowledge, this is one of the first studies in Iran to investigate SQ among community-dwelling adults. The findings of this study indicated that the majority of participants have poor SQ, and several factors were related to SQ. Furthermore, among the various components of SQ, the participants demonstrated better sleep efficiency.

 The present study found that approximately three-quarters of the participants have poor SQ. The prevalence of poor SQ in this study is higher than that reported in some previous studies. For example, a study by Sun et al in China found that 43.6% of the participants have SQ.^30^ Moreover, studies conducted in Thailand^31^ and China^32^ reported a lower prevalence of poor SQ (44% and 29.57%, respectively) than the proportion observed in the present study. However, a study by Malakouti et al conducted on Iranian individuals aged over 60 years found that more than 80% of the participants have poor SQ.^33^ However, this study found a very high prevalence of poor SQ, which requires further explanation. These differences may be attributed to variations in the demographic and health condition of study participants as well as differences in cultural and lifestyle factors. A likely explanation is that the majority of participants were either retired or unemployed, which may have contributed to an inactive lifestyle and feelings of low self-worth. These factors could have potentially led to the development of mental health issues such as depression and anxiety, which are known to negatively impact SQ.^34,35^ On the other hand, the majority of participants reported being in the middle or lowest financial level. This unsatisfactory financial situation could have contributed to emotional and mental health problems, which could negatively affect SQ.^36,37^ Additionally, poor health conditions and co-morbidities in older adults are known to be associated with poor SQ. Among the participants, the prevalence of diseases that affect SQ such as musculoskeletal pain, diabetes, and cardiovascular disease was relatively high.^38-40^

 Regarding sleep components, the mean scores of “sleep efficiency” were higher than other components. This finding suggests that the participants were able to use their time in bed more efficiently and were less likely to experience difficulties falling asleep or staying asleep. However, it is important to note that this finding is inconsistent with the results of other studies that have displayed “sleep latency” as the most common sleep problem among the older adults.^31,41^ The results also revealed that “use of sleep medication” has the lowest mean score. This could be because older adults may consider a decrease in SQ a natural part of the aging process and therefore may not report it to their doctor. Additionally, cultural attitudes toward sleep medication may be negative among participants, which could also contribute to their reluctance to use medication to improve their SQ.

 The present study found that females have significantly poorer SQ compared to males. This is consistent with previous epidemiological studies, which have shown that being female is an independent risk factor for sleep disorders.^42,43^ One of the reasons that may affect SQ in females is hormonal changes that occur during menopause.^44^ Levels of estradiol decrease during and after menopause, while levels of follicle-stimulating hormone increase. These hormonal changes can lead to problems with sleeping; as a result, SQ may decrease.^45^ In addition, there are differences in comorbidities between males and females.^46^

 Self-rated health was significantly related to SQ, which is consistent with the findings of previous studies.^47^ These results suggest that more attention should be given to improving the SQ of middle-aged and older adults who perceive their health as poor since they may be experiencing physical or psychological symptoms that negatively impact their SQ.^48^ Many people who suffer from chronic pain, depression, anxiety, or other health issues have trouble falling asleep or staying asleep. This can make their health problems worse or lead to new ones. Poor SQ can also affect how they feel about their health.

 Interestingly, the study did not find a statistically significant difference between respiratory diseases and SQ, which differs from the findings of other studies.^49^ A possible explanation for this might be that respiratory diseases were assessed by self-report in this study; therefore, it is possible that the participants may not have been entirely aware of their breathing problems, which could have affected the results.

 This study revealed that SQ is related to depression and anxiety, which is consistent with the findings of the previous study.^21^ There may be various reasons to explain the link between poor SQ and depression and anxiety. One possible reason for this is the existence of an overlap in the abnormalities of neurotransmitters and brain structures involved in problems related to the sleep-wake cycle, anxiety, and depression.^50-54^ Furthermore, individuals with depression tend to have constant worry and rumination, which can lead to a pessimistic interpretation of existing conditions and negatively impact their ability to sleep.^55^ A study by Kaneita et al demonstrated that there are common factors that independently affect sleep, anxiety, and depression.^56^ These common factors may influence the relationship between SQ and depression and anxiety. Although the current study was adjusted for the effect of confounding variables, some remaining unmeasured confounding factors may still affect the relationship between perceived SQ with depression and anxiety.

 The present study evaluated a relatively large randomly selected sample using validated measures of SQ. However, it is important to note that this study has some limitations. Firstly, the cross-sectional design of the study did not allow for conclusions about the temporal relationships between SQ and its related factors. Secondly, the use of self-reported data may introduce reporting bias, which could lead to misclassification of some participants. However, it is worth noting that self-reported data is a common and widely used method of data collection in sleep research. Despite these limitations, this study provides valuable insights into the factors associated with SQ among middle-aged and older adults and highlights the importance of addressing SQ in this population.

**Highlights**

- In Ardakan, Iran, 76.36% of the population above 50 had poor sleep quality (SQ).
- The use of sleep medication has the lowest score compared to other components of the Pittsburgh Sleep Quality Index (PSQI).
- Sleep efficiency was the most common sleep problem among participants.
- Depression and anxiety were factors related to SQ in adults aged ≥ 50.

## Conclusion

 The findings of this study demonstrate that the prevalence of poor SQ is high among Iranian middle-aged and older adults. To improve SQ, it may be necessary to implement interventions that address the specific factors identified in this study such as health conditions, improving overall health perceptions, depression, and anxiety. Overall, these findings highlight the need for interventions aimed at improving SQ among middle-aged and older adults in Iran and other populations around the world.

## Acknowledgements

 The authors wish to acknowledge the study participants, the staff at ACSA, and the Research Center on Aging personnel. The authors also appreciate the University of Social Welfare and Rehabilitation Sciences for supporting this study.

## Authors’ Contribution

**Conceptualization:** Ahmad Delbari, Fatemeh Ghavidel.

**Data curation:** Elham Hooshmand.

**Formal analysis:** Mohammad Bidkhori.

**Funding acquisition:** Ahmad Delbari.

**Investigation:** Fatemeh Ghavidel, Sara Efati.

**Methodology:** Fatemeh Ghavidel, Mohammad Saatchi.

**Resources:** Elham Hooshmand, Mohammad Saatchi.

**Validation:** Yadollah Abolfathi Momtaz.

**Visualization:** Mohammad Bidkhori.

**Writing–original draft:** Ahmad Delbari, Fatemeh Ghavidel.

**Writing–review & editing:** Yadollah Abolfathi Momtaz, Fatemeh Ghavidel, Mohammad Bidkhori.

## Competing Interests

 The authors have no relevant financial or non-financial interests to disclose.

## Funding

 The Iranian Ministry of Health and Medical Education has contributed to the funding used in the PERSIAN cohorts by grant number 700/151. Furthermore, this work was supported and funded by the University of Social Welfare and Rehabilitation Sciences.
